# The Role of Selection and Migration in the Evolution of (Auto)Immunity Genes

**DOI:** 10.1007/s00239-024-10182-z

**Published:** 2024-06-26

**Authors:** Konstantinos Voskarides

**Affiliations:** 1 Department of Basic and Clinical Sciences, University of Nicosia Medical School, Nicosia, Cyprus; 2 School of Veterinary Medicine, University of Nicosia, Nicosia, Cyprus

**Keywords:** Antagonistic pleiotropy, Autoimmune disease, Adaptation, Evolutionary medicine, Mutation, Migration

## Abstract

The genetic architecture of multiple sclerosis is complicated. Additionally, the disease incidence varies per population or per geographical region. A recent study gives convincing explanations about the north–south incidence gradient of multiple sclerosis in Europe, by analyzing ancient and modern human genomes. Interestingly, the evidence shows that multiple sclerosis associated immunogenetic variants underwent positive selection in Asian and European populations. Lifestyle and pathogen infections probably shaped the overall multiple sclerosis risk. These results complete the findings of previous studies that showed that a high percentage of the autoimmunity associated genetic variants are under selection pressure.

Multiple migration routes have shaped the present-days *Homo sapiens* populations. The genetic structure of the European populations has been mainly formed by three prehistoric migration waves. The first one is related with the arrival of hunter-gatherers approximately 45,000 years ago, the second one with the expansion of the Middle East farmers during the Neolithic era, about 11,000 years ago, and the last one with the pastoralists coming from the steppes of Western Asia, about 5000 years ago (Lazaridis et al. 2014; Posth et al. 2023; Reardon 2024; Irving-Pease et al. 2024). In January of 2024, four landmark papers have been published in *Nature*, giving important insights about the genetic characteristics of the European populations (Allentoft et al. 2024a, b; Barrie et al. 2024; Irving-Pease et al. 2024). In this commentary, I discuss the importance of the findings of one of these studies by Barrie et al. (2024). The authors of this paper give a reliable explanation about the high incidence of multiple sclerosis in the Northern European populations by analyzing multiple ancient and present-day human genomes.

The predisposition architecture of multiple sclerosis is complicated. Genome wide association studies (GWAS) have identified many genetic variants that predispose for this multifactorial disease (Patsopoulos et al. 2019). The environmental parameter is not clear; however, it is well known that Epstein–Barr virus infection plays an important role for the disease predisposition (Bjornevik et al. 2022). Genetic variants exhibiting strong predisposition for multiple sclerosis are found inside the highly polymorphic HLA genes. Interestingly, carriers of the HLA-DRB1*15:01 variant have approximately a threefold increase for multiple sclerosis risk (Greer 2015).

Barrie et al. 2024, investigated the ancestry of 410,000 genomes registered in the UK Biobank (Bycroft et al. 2018), using as a reference panel 318 ancient DNA samples. They also included in their analysis 86 new genomes of ancient individuals dated from the Medieval and post-Medieval periods from Denmark, representing a typical Northern European population, plus 1664 previously published ancient genomes. They managed to cluster the ancient genomes in certain ancestries: western hunter-gatherers (WHG), eastern hunter-gatherers (EHG), Caucasus hunter-gatherers (CHG), farmers (Anatolian (ANA) + Neolithic), steppe and African. Additionally, they identified a “typical ancestral background” for each UK Biobank DNA sample. According to these data, they found that the frequency of the HLA-DRB1*15:01 variant is highest in modern populations from Finland, Sweden, and Iceland and it was high in ancient populations of steppe ancestry.

Barrie et al. (2024), used special statistical tests that are based on the length of haplotypic blocks (linkage disequilibrium) to examine the genetic origin of genetic variants associated with multiple sclerosis, found in the present-day European genomes. They concluded that the present-day multiple sclerosis genetic variants originate almost exclusively from the steppe pastoralists populations, about 5000 years ago, migrated in the Northern regions of Europe. This can explain the north–south gradient of multiple sclerosis incidence that is observed today. The single nucleotide polymorphism (SNP) rs3129934 that is found in linkage disequilibrium with the HLA-DRB1*15:01 variant seems to have the largest change of genetic risk over time (selection coefficient = 0.018). For the purpose of this commentary article, it was investigated if the genetic position of rs3129934 is included in any of the candidate human genetic regions under selection, listed in the PopHumanScan database (Murga-Moreno et al. 2019). The PopHumanScan database includes candidate human genetic regions being under selective pressure, based on the data derived from the 1000 Genomes Project (Auton et al. 2015). The 1000 Genomes Project produced physical and analytical genetic maps through the genotyping of millions of SNPs in individuals coming from 26 different human populations. Interestingly, the genetic variants rs3129934 and HLA-DRB1*15:01 are found inside a genetic region with strong evidence for selection (Fig. 1). It is even more interesting that the Finnish population displays the highest evidence for selection for this region, according to the Fu and Li's D selection test (Fu and Li 1993).

**Fig. 1:**
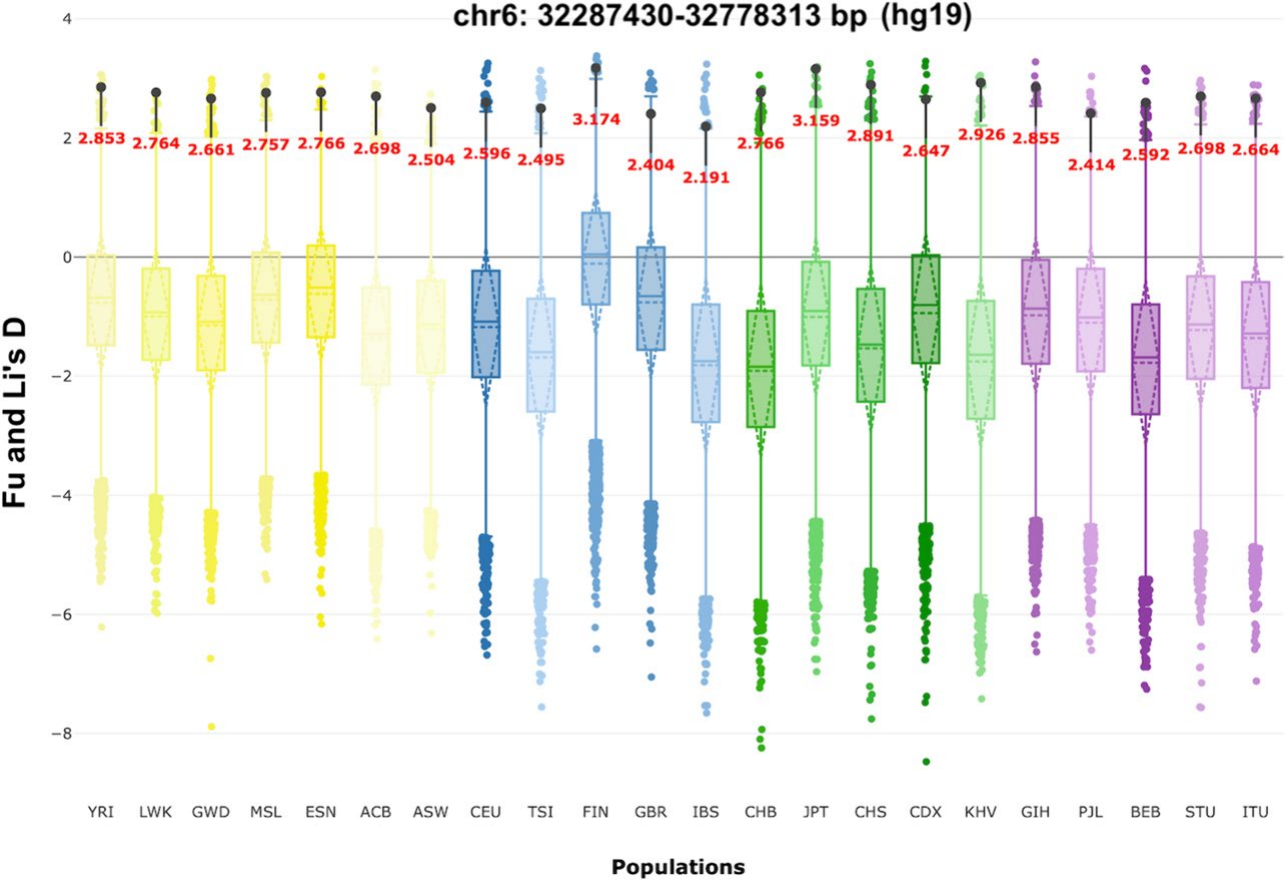
Fu and Li's D values for the 1000 Genomes Project populations (populations codes are found below), in a candidate genetic region under selection found in the HLA region of chromosome 6 (see text for details). Values that significantly deviate from zero are informative about distinct demographic and/or selective events (Fu and Li 1993). The Finnish population displays the highest value. The graph has been created by the PopHumanScan database (Murga-Moreno et al. 2019). East Asia: CHB: Han Chinese, JPT: Japanese, CHS: Southern Han Chinese, CDX: Dai Chinese, KHV: Kinh Vietnamese. Europe: CEU: Utah residents with Northern and Western European ancestry, TSI: Tuscan, GBR: British, FIN: Finnish, IBS: Spanish. Africa: YRI: Yoruba in Nigeria, LWK: Luhya in Kenya, GWD: Gambian, MSL: Mende in Sierra Leone, ESN: Esan in Nigeria, ASW: African-American, ACB: African-Caribbean. South Asia: GIH: Gujarati Indian in Houston, PJL: Punjabi in Pakistan, BEB: Bengali in Bangladesh, STU: Sri Lankan, ITU: Indian

There is reliable evidence that pathogen infections have contributed to the evolution of genes related with the immune system (Kerner et al. 2021, 2023). Barrie et al. 2024, hypothesize that genetic variants that predispose for multiple sclerosis had a protective role against zoonoses, protecting the farmers for the last 10,000 years. They conclude that the north–south gradient of multiple sclerosis prevalence in Europe resulted from the combination of two events: (a) the steppe pastoralist migration in the Northern Europe, (b) the positive selection of particular HLA variants that were transferred by migrations in the Northern regions of Europe. Diseases that exhibit special geographical patterns is not something unusual in genetics. Sickle cell disease is the best-known example. The prevalence of sickle cell disease is high in geographical regions with a high frequency of *Plasmodium falciparum* infections, known as malaria (Kariuki and Williams 2020). The reason is that carriers of the sickle cell disease mutation are resistant for this infection. Similar explanations have been given for carriers of alpha-thalassemia, beta-thalassemia and G6PD deficiency (Kariuki and Williams 2020).

Another recent publication in *Nature Communications* (Pankratov et al. 2022) gives more evidence for a positive selection pressure on genetic variants that predispose for autoimmune diseases. Pankratov et al. 2022, proved that 28% of the known risk loci in 21 inflammatory diseases show evidence for weak and moderate positive selection. Part of those events seem to be population specific. In addition, they showed that the frequency of a significant percentage (19%) of the risk loci under selection has been increased because they are found in linkage disequilibrium with the actual selection targets. The function prediction of the disease risk loci under selection shows a close association with the enhancement of immunity pathways’ activation. These pathways, like T cell activation and positive regulation of cytokine production, are crucial for a variety of pathogen infections.

Positive selection of disease risk genetic variants that influence the immune response is closely related with the antagonistic pleiotropy phenomenon, firstly described by George Williams (Williams 1957). George Williams was also one of the first evolutionary biologists that linked health with evolution. Today, a whole scientific field is currently expanding worldwide, known as Evolutionary Medicine (Stearns 2020; Voskarides 2020; Perry 2021). Antagonistic pleiotropy proposes that genetic variants that contribute to aging or age-related degenerative diseases are probably under selection since they increase fitness at a young age. An extended and detailed discussion on multiple examples of antagonistic pleiotropy in humans can be found in the review article by Byars and Voskarides (2020). Autoimmune diseases can of course onset at an early age, but the principle of the dual nature of genetic variants remains the same.

Concluding, contagious diseases can dramatically change the human genetic pool. An extreme example is bubonic plague (known as black death) that killed about 50% of the fourteenth century European population (Klunk et al. 2022; Hui et al. 2024). We can imagine that the survivors, the other 50%, probably carried some immunity related protective variants. Even the recent example of COVID-19 infection may have contributed to frequency alterations of some immune genetic variants. Large scale population-wide genomic studies in the near future will shed more light on these exciting aspects of population genetics.
